# Description of *Skrjabinoclava pharyngophila* n. sp. (Nematoda: Acuariidae) from the oropharynx of the sacred kingfisher *Todiramphus sanctus* (Vigors & Horsfield) in New Zealand

**DOI:** 10.1007/s11230-026-10285-9

**Published:** 2026-05-27

**Authors:** B. Presswell, P. Dorle, I. Scott, J. Bennett

**Affiliations:** 1 Department of Zoology, University of Otago, PO Box 56, Dunedin, New Zealand; 2 Birdcare Aotearoa, 74 Avonleigh Rd. Green Bay, Auckland, New Zealand; 3 School of Veterinary Science, Massey University, Private Bag 11222, Palmerston North, 4410 New Zealand

## Abstract

The sacred kingfisher *Todiramphus sanctus* is a widespread bird species that is non-migratory in New Zealand, in contrast to elsewhere in the world. We describe a new species of *Skrjabinoclava*, a genus of acuariid nematodes, recovered from the oropharynx of sacred kingfishers in New Zealand. This is the first record of *Skrjabinoclava* in Australasia and the first publicly available DNA sequence for the genus. *Skrjabinoclava pharyngophila* **n. sp.** is unique in its infection site, occupying the oropharyngeal mucosa, which is atypical for *Skrjabinoclava* species, which usually infect the proventriculus or oesophagus. Morphological and molecular analyses confirm the distinctiveness of this new species, with DNA sequencing placing it within the family Acuariidae. The prevalence of infection was high in North Island birds, while none were found in South Island birds, suggesting the parasite is geographically restricted, possibly due to the distribution of intermediate hosts or the sedentary behavior of the host population. The presence of the parasite in New Zealand sacred kingfishers and its unique infection site offers new insights into the evolution of parasitism in isolated populations.

## Introduction

The sacred kingfisher *Todiramphus sanctus* (Vigors & Horsfield) (previously *Halcyon sancta*) is widespread across coastal and woodland habitats of Australia and New Zealand, and also occurs on several islands in the western Pacific and Southeast Asia. In the majority of its range, it is seasonally north-south migratory. In New Zealand, however, the species remains year-round but exhibits altitudinal migration, with inland-breeding birds (up to 700 m a.s.l.) moving to coastal areas during winter (McKinlay, 2013). There is no evidence of regular movement between the North and South Islands, suggesting that populations are otherwise sedentary. Consequently, parasites of *T. sanctus* in New Zealand are likely to be geographically restricted, in contrast to those of long-distance migratory hosts that disperse widely along flyways. For instance, the nematode *Skrjabinoclava sealyi* Anderson & Wong, 1992 occurs in the ringed plover *Charadrius hiaticula hiaticula* L., which migrates between the United Kingdom, Iceland, Greenland and northeastern Canada (Anderson & Wong, 1992). Such migratory movements facilitate the intercontinental dispersal of their parasites, whereas parasites of resident hosts, such as *T. sanctus* in New Zealand, are more likely to represent endemic lineages. The only record of a helminth parasite from *T. sanctus* in New Zealand is that of the nematode *Porrocaecum reticulatum* Linstow, 1899 (Bennett et al., 2022). However, several helminths have been recorded from the same host in Australia. Mutafchiev (2016) described two species of *Quasithelazia* Maplestone, 1932 (*Q. pearsoni* Mutafchiev, 2016 and *Q. minuta* Mutafchiev, 2016), acuariid nematodes from *T. sanctus* in Queensland and South Australia. Mawson (1968) established the hartertiid genus *Alainchabaudia* Mawson, 1968 for nematodes from coraciiform birds, including *A. alcedinis* Mawson, 1968 from *T. sanctus* in Queensland and South Australia. Air-sac nematodes of the genus *Serratospiculum* Skrjabin, 1915 have also been reported from kingfishers in Australia (Reece et al., 1992). In addition, plerocercoids of the cestode *Spirometra erinacei* (Rudolphi, 1819) have been found in this host (Reece et al., 1992), and the acanthocephalan *Centrorhynchus horridus* (Linstow, 1897) has been recorded from New Britain, New South Wales, New Caledonia, and Queensland (Linstow, 1897; Johnston, 1910; Porta, 1913; Johnston & Edmonds, 1948).

At present, 31 species are recognised within the acuariine genus *Skrjabinoclava* (WoRMS, accessed December, 2025). Most species are parasitic in the proventriculus of birds, particularly waders (Charadriiformes). Two species have been reported from the oesophagus: *Skrjabinoclava halcyoni* Ryzhikov & Khokhlova, 1964, described from the black-capped kingfisher *Halcyon pileata* (Boddaert) in Vietnam, and *S. horrida* (Rudolphi, 1809), originally described from “*Scolopacis gallinulae*”—probably the jack snipe *Lymnocryptes minimus* Brünich—and presumed to be of European origin. However, comparison with *S. horrida* is complicated, as this name has been applied broadly to specimens from a wide range of hosts and localities worldwide. Either *S. horrida* exhibits exceptional ecological plasticity, or many of these records represent misidentifications. Although later reviews list the oesophagus as a site of infection for the species, these reports merely reiterate Rudolphi’s (1809) original observation, and we have found no convincing new records of the species from that anatomical site. Furthermore, records of *S. horrida* from kingfishers have been questioned; Wong & Anderson (1987) considered these more plausibly referable to *S. halcyoni*. There are no previous records of any *Skrjabinoclava* species utilising the pharynx as infection site.

In this paper we describe a new species of *Skrjabinoclava* recovered from the oropharynx of sacred kingfishers *T. sanctus* from North Island, New Zealand, which we name *Skrjabinoclava pharyngophila* **n. sp.** This species represents the first record of *Skrjabinoclava* from Australasia, and the first from this host, as well as a unique infection site in the host.

## Materials and Methods

*Collection of material*

Fourteen deceased sacred kingfishers (*Todiramphus sanctus*) were donated by the Dunedin Wildlife Hospital (South Island, New Zealand) and six from BirdCare Aotearoa (Auckland, North Island), which also provided nematodes extracted from the oropharynx of three live birds. South Island specimens were collected between May 2019 and April 2025, and all North Island samples were from 2025. The carcases were stored frozen and thawed prior to examination. The buccal mucosa was thoroughly examined for the presence of parasites, and the gastrointestinal tract was removed for further investigation.

*Morphological analysis*

Nematode specimens were temporarily mounted in lactophenol for light microscopy and photography. Measurements were taken from digital images using Fiji software (Schindelin et al., 2012), based on photographs captured with an Olympus CH-2 compound microscope equipped with a MD500L camera. All measurements are given in micrometres unless otherwise indicated. The range is followed by the mean in parentheses. Drawings were prepared from focal series of microphotographs, and from slides using a microscope-mounted drawing tube.

Four specimens were processed for scanning electron microscopy (SEM). They were fixed in 2.5% glutaraldehyde in 0.1 M cacodylate buffer, post-fixed in 1% osmium tetroxide, dehydrated through an ethanol series, critical-point dried using carbon dioxide, and sputter-coated with gold/palladium (60:40) to a thickness of approximately 10 nm. The coated specimens were examined using a JEOL 6700F field-emission scanning electron microscope (JEOL Ltd., Tokyo, Japan) at the Otago Centre for Electron Microscopy, University of Otago, New Zealand.

*Molecular analysis*

Genomic DNA was extracted from two specimens using DNeasy Blood & Tissue Kit (Qiagen, Hilden, Germany) according to the manufacturer’s protocol. Polymerase chain reactions (PCRs) were performed, targeting the small ribosomal subunit 18S gene using primers Nem18SF and Nem18SR (Wood et al., 2013). PCR conditions followed that of Bennett et al. (2022). Positive PCR products were cleaned using EXOSAP-TMTM Express PCR Product Cleanup Reagent (USB Corporation, Cleveland, OH, USA), following manufacturer’s instructions. Sanger sequencing by capillary electrophoresis was performed by the Genetic Analysis Service, Department of Anatomy, University of Otago (Dunedin, New Zealand). Sequences were imported into Geneious Prime® 2026.0.2 (Kearse et al., 2012), trimmed using the trim function with default parameters, and manually edited for incorrect or ambiguous base calls. The resulting sequence was submitted to GenBank under accession number PZ305674-5.

To infer the phylogenetic position of the new *Skrjabinoclava* species within the context of Family Acuariidae we produced a Bayesian inference tree in Geneious Prime with the MrBayes Plugin (v. 2.2.4) (Huelsenbeck & Ronquist, 2001). We downloaded available sequences from GenBank within Infraorder Spiruromorpha. Outgroups were selected to root phylogeny within Acuariidae, and so species of Superfamily Aproctoidea were selected. The model of evolution was determined using jModelTTest2 v.2.0 (Darriba et al., 2012; Guindon & Gascuel, 2003) in the Cyberinfrastructure for Phylogenetic Research CIPRES (Miller et al., 2010); estimated to be GTR + I using Akaike information criteria (AIC) model selection. Analysis performed had random starting trees for two runs (each with one cold and three heated chains), employing a Markov Chain Monte Carlo approach for sampling the joint posterior probability distribution across 10,000,000 generations at a heating chain temperature of 0.02. The first 25% of samples were discarded as burn-in. Mixing and convergence estimates were evaluated through MrBayes output files to ensure resulting phylogeny was appropriately estimated. The resulting tree was summarised in a 50% majority-rule consensus tree with clade credibility support values (Bayesian posterior probability, BPP) and branch length information. BPP higher than 0.8 was considered moderately supported and greater than 0.95 was considered high support for nodal positions. Uncorrected pairwise genetic distances were estimated in MEGAv11.0.3 (Tamura et al., 2021).

## Results

**NEMATODA Diesing, 1861**

**Acuarioidea Railliet, Henry & Sisoff, 1912**

**Acuariidae Railliet, Henry & Sisoff, 1912**

**Acuariinae Railliet, Henry & Sisoff, 1912**

***Skrjabinoclava*** **Sobolev, 1943**

***Skrjabinoclava pharyngophila*** **Presswell & Bennett n. sp.**

### *Description* (Figs. 1a-g; 2a-d; 3)

**Fig. 1.**
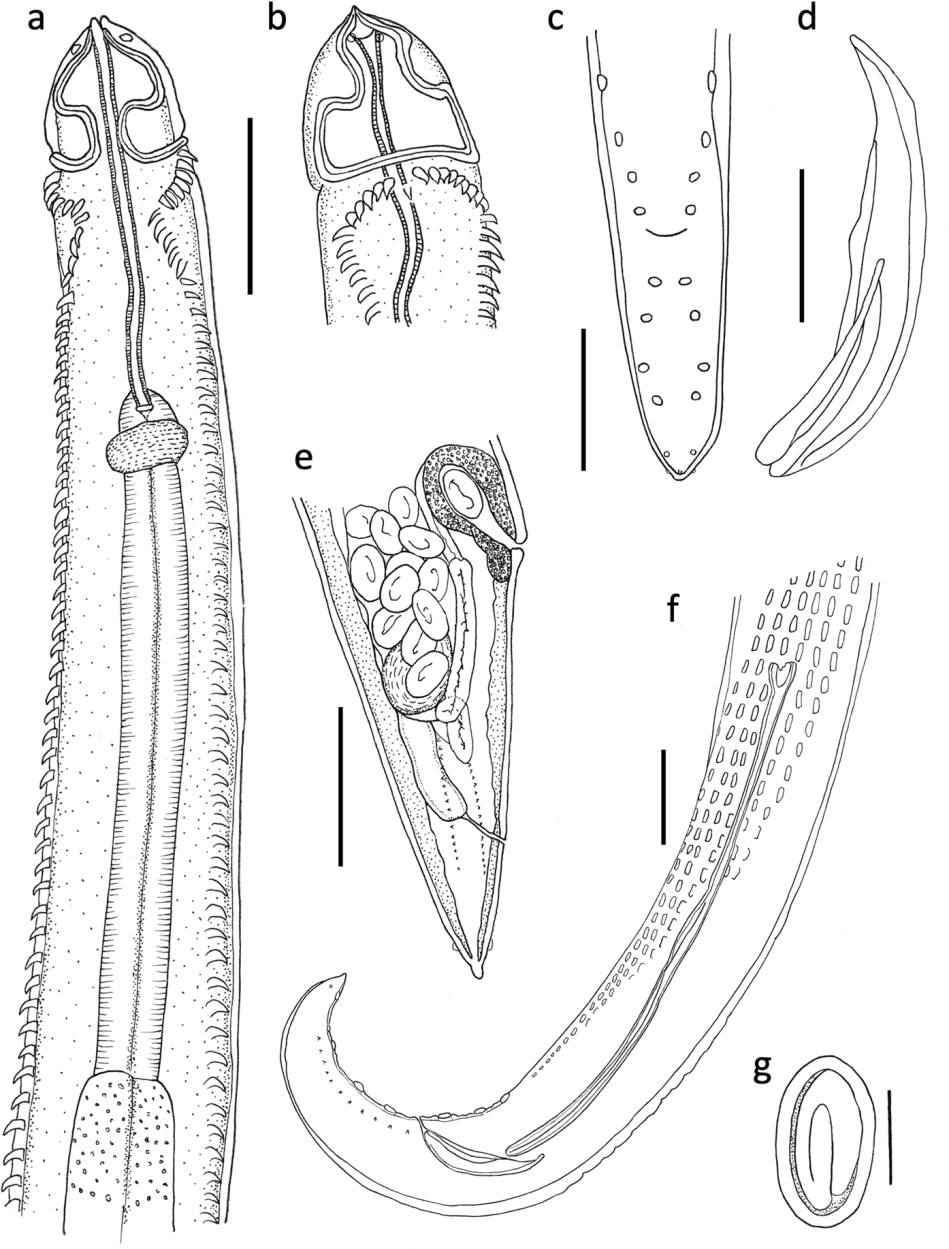
Line drawings of *Skrjabinoclava pharyngophila* **n. sp.** **a** Female, anterior end, dorsal view. **b** Female, anterior end, lateral view. **c** Male, position of caudal papillae, **d** Male, right spicule. **e** Female, posterior end, f) Male, posterior end, g) egg. Scale bars: **a**, **b**, **c**, **e** and **f** 100 µm; **c** 50 µm; **g** 20µm.

Based on observations of 15 cleared specimens, and 2 specimens photographed using SEM.

*General*

Medium sized worms with cordons slightly longer than wide, recurrent, with cuticular collar posterior to cordons visible in some specimens (Fig. 1a, b, 2a). Anterior with two lateral pseudolabia, each bearing cuticular tooth with two cephalic papillae and an amphid. Buccal cavity not sharply delineated from pharynx; pharynx long, with strongly delineated transverse striations. Nerve ring slightly posterior to junction of pharynx and oesophagus (Fig. 1a). Excretory pore ventral, slightly posterior to nerve ring. Muscular oesophagus short, glandular oesophagus long. Deirids with single pointed tip, located laterally, slightly posterior to cordons (Fig. 1b). Body spines forming incomplete arch immediately anterior to deirids; seven spines in oblique row then turning posteriorly to form two parallel sublateral rows, constricted anteriorly. Anterior spines 20-25 long; decreasing in size posteriorly, continuing almost to posterior end (Fig. 1a, b).

**Fig. 2.**
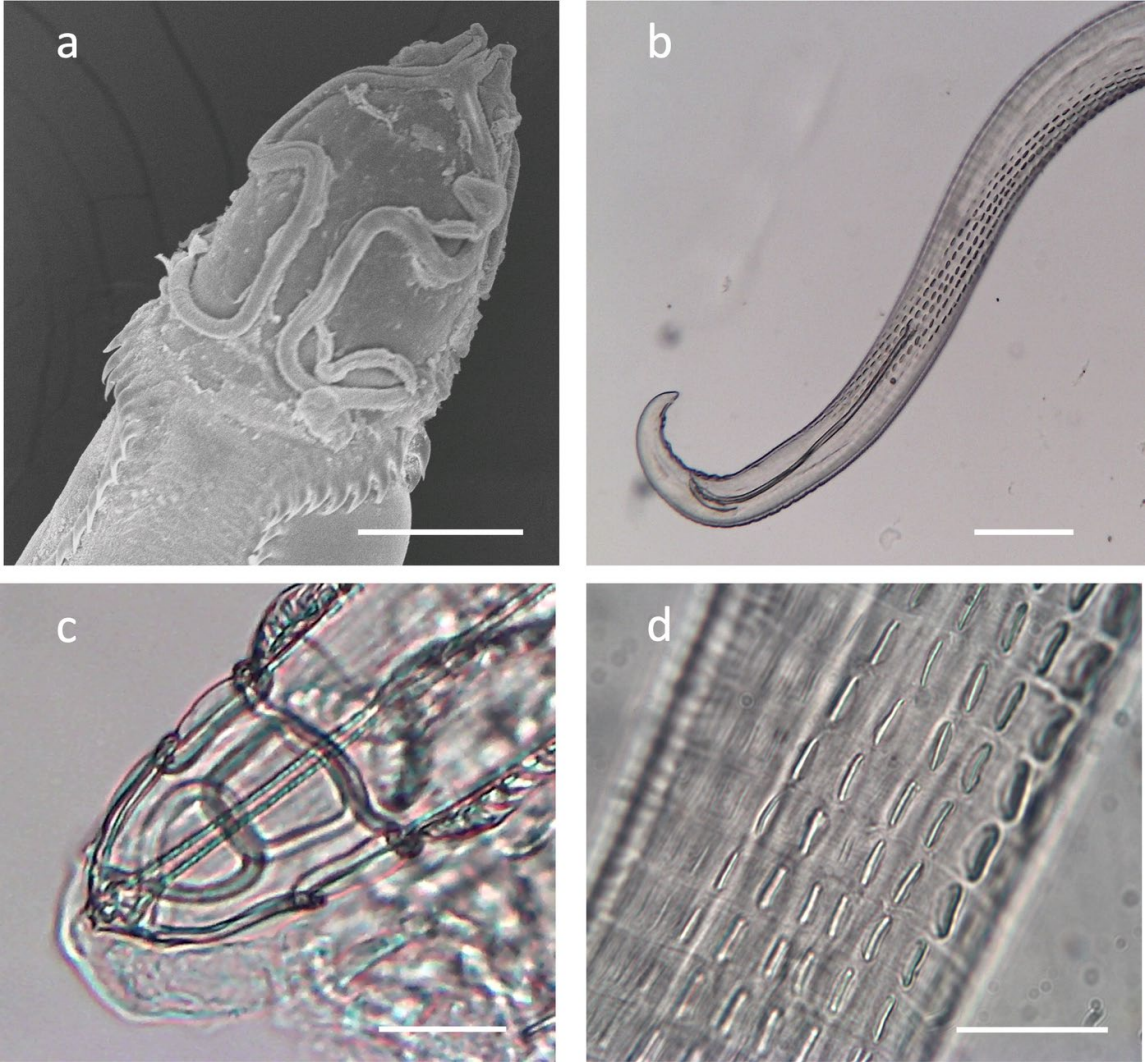
**a** Scanning electron micrograph of head end of *Skrjabinoclava pharyngophila* **n. sp**., dorsal view. **b** Light micrograph of male posterior end with torsion, showing spicules, papillae and area rugosa. **c** Head end of specimen showing ‘pileus’ or transparent cap in which the worm attaches to the host. **d** Closeup of area rugosa. Scale bars: **a** 50µm, **b** 200µm, **c** and **d** 50µm

*Male*

Male (N = 7). Body length 5.6–7.6 (6.5) mm. Greatest width at mid-body 165–209 (186); body width at cloaca 66-78 (72). Pharynx 221-278 (241) in length. Nerve ring 234–269 (255), excretory pore 285–332 (305) and deirids 97–117 (110) from anterior extremity. Cordon field 86–110 (98) in length. Muscular oesophagus 381–558 (453) long x 43-54 (47) wide, on average 18% length of glandular oesophagus, 1.87–2.9 (2.6) mm long x 89-135 (106) wide. Spicules dissimilar and unequal. Right spicule broad and falciform; proximal end pointed, distal end rounded; convex margin reinforced by a longitudinal rib, concave margin with a secondary rib traversing the blade and terminating at mid-length; 98–129 (114) in length (Fig. 1d, f, 2b). Left spicule long, slender and arcuate, with funnel-shaped proximal end; shaft twisting longitudinally at mid-length, distal portion appearing duplicated or bilaminate, the elements remaining fused throughout; 312–506 (435) (Fig. 1f, 2b). Tail 145–182 (161) in length. Area rugosa consisting of 6 parallel rows of cuticular ridges, located in posterior third of body; extending 25-30% body length (Fig. 1f, 2b, d). Caudal extremity curved or coiled ventrally, with very narrow caudal alae bearing 4 pre-anal and 5 post-anal pairs of short pedunculate papillae, plus two small sessile papillae ventral to posterior pair. Phasmids near tip of tail (Fig. 1c).

*Female*

Female (N = 8). Body length 5.6–8.4 (7.1) mm. Greatest width at anterior extent of uterus 220–296 (262); body width at vulva 136-189 (152), body width at anus 58-87 (67). Pharynx 228–295 (266) in length. Nerve-ring 236–321 (288), excretory pore 290–362 (330), and deirids 115–145 (136) from anterior extremity. Cordon field 122–135 (129) in length. Muscular oesophagus 267-510 (400) long x 47-51 (49) wide, on average 16% length of glandular oesophagus, 1.8–3.4 (2.6) mm long x 101-147 (125) wide. Vulva near posterior end of body, 278–383 (325) from tip of tail. Monodelphic uterus, loops posterior to vulva in mature specimens. Vagina surrounded by thick muscle fibres, divided into vagina vera (76-85 long, n=2) and vagina uterina (112 long, n=1) (Fig. 1e). Eggs larvated; 35–42 (39) x 21–24 (23) (Fig. 1e, g). Anus 78–112 (99) from tip of tail. Phasmids present at tip of tail. (Fig. 1e).

*Taxonomic summary*

*Type host.* Sacred kingfisher *Todiramphus sanctus* (Vigors & Horsfield)

*Type locality.* Laingholm, Waitākere, North Island, New Zealand (46°26’S 168°130 E).

*Other locality.* Auckland region (Bethells Beach, Te Atatū, Whitford, Titirangi, Parau), North Island, New Zealand.

*Site of infection.* Cephalic region embedded in oropharyngeal membrane, with body extending into oral cavity, or under the membrane in a cyst-like formation.

*Prevalence.* 100% (N = 6) birds from North Island. None found in birds from South Island.

*Intensity.* Range 2–16, average 5.8.

*Type material*. Holotype (male) W.003991, allotype (female) W.003992 paratypes (2 male, 2 females) W.003993 (in ethanol), deposited with the Museum of New Zealand, Te Papa Tongarewa, Wellington, NZ.

*GenBank accession number:* 18S gene: PZ305674-5.

*Zoobank reference:* urn:lsid:zoobank.org:act:B56DF0D3-FF39-4763-A7BC-E4C1F34067C4

*Etymology.* The species name *pharyngophila* is an adjective formed from the Greek *pharynx* (throat) and *philos* (loving), with the feminine ending *-a* to agree with the feminine genus *Skrjabinoclava*. The name refers to the nematode’s unusual occurrence in the pharyngeal region of its host.

### **Remarks**

The specimens from *Todiramphus sanctus* conform to the diagnosis of *Skrjabinoclava* (Anderson & Wong, 1992), in having paired pseudolabia with two pairs of cephalic papillae and a pair of amphids; cordons laterally recurrent; pharynx elongate, lined with transversely striated cuticle; body spines forming an arch immediately posterior to cordons, continuing posteriorly as two parallel rows, decreasing in size; deirids inconspicuous, situated beneath spine arch; males with area rugosa; caudal alae narrow, with four pairs of preanal and five pairs of postanal papillae; spicules unequal and dissimilar; vulva slightly anterior to anus.

The cordon configuration of *S. pharyngophila* **n. sp.** is most similar to that of *S. amaurornae* Schmidt & Kuntz, 1972, *S. bartlettae* Wong & Anderson, 1988*, S. halcyoni*, *S. pusillae* Wong & Anderson, 1987, *S. rallae* Schmidt & Kuntz, 1972, and *S. wilsonae* Wong & Anderson, 1987. However, the new species is larger than all of these taxa except *S. halcyoni*. Each of the other species can be distinguished from the new species by a combination of characters, including overall body size, body-spine arrangement, and the shape of the right spicule, among other features. For example, in *S. amaurornae*, the longitudinal rows of spines flare proximally and the left spicule has a distinctly hooked tip. *S. bartlettae* is characterised by a rounded cuticle surrounding the left spicule and a “nipple-like” tip of the right spicule. In *S. pusillae*, the right spicule has a distal end described as flattened, resembling the head of a tomahawk. *S. rallae* possesses a right spicule with a strong ventral apical process and a sharply pointed left spicule. In *S. wilsoniae*, the body spines do not decrease in size posteriorly and the right spicule has a cone-shaped, ventrally directed distal end.

Morphologically, the new species is clearly most closely related to *S. halcyoni*, a parasite of kingfishers from several regions worldwide. *Skrjabinoclava halcyoni* has been recorded from the black-capped kingfisher *Halcyon pileata* in Vietnam (Ryzhikov & Khokhlova, 1964; type host), and from the white-throated kingfisher *H. smyrnensis* (L.) in China (Hsü, 1957) and India (Singh, 1948), the latter records originally described as *S. cincli* and *S. horrida* respectively, but subsequently reassigned to *S. halcyoni* by Schmidt & Kuntz (1972). However, detailed morphological and morphometric data for *S*. *halcyoni* are available only from the original description by Ryzhikov & Khokhlova (1964), which was based on a single male and female.

The male of *S. halcyoni* described by Ryzhikov & Khokhlova (1964) was slightly smaller than males of the present species (5.4 mm versus 6.0–7.6 mm), with most measurements correspondingly smaller. Consistent with this size difference, the left spicule of *S. halcyoni* is shorter (360 µm versus 379–506 µm in the new species), although the right spicule falls within a similar range (109 µm versus 98–129 µm). As a result, the spicule length ratio differs markedly between the two species (22% in *S. pharyngophila* versus 30% in *S. halcyoni*). Females of the two species are within the same size range, but differ in the length of the tail posterior to the vulva (400 versus 278-383 (325.) in *S. pharyngophila*) and anus (132 versus 78-112 (99) in *S. pharyngophila*), and in having larger eggs (43-46 x 26-30 versus 35-42 x 21-24 in *S. pharyngophila*).

**Fig. 3.**
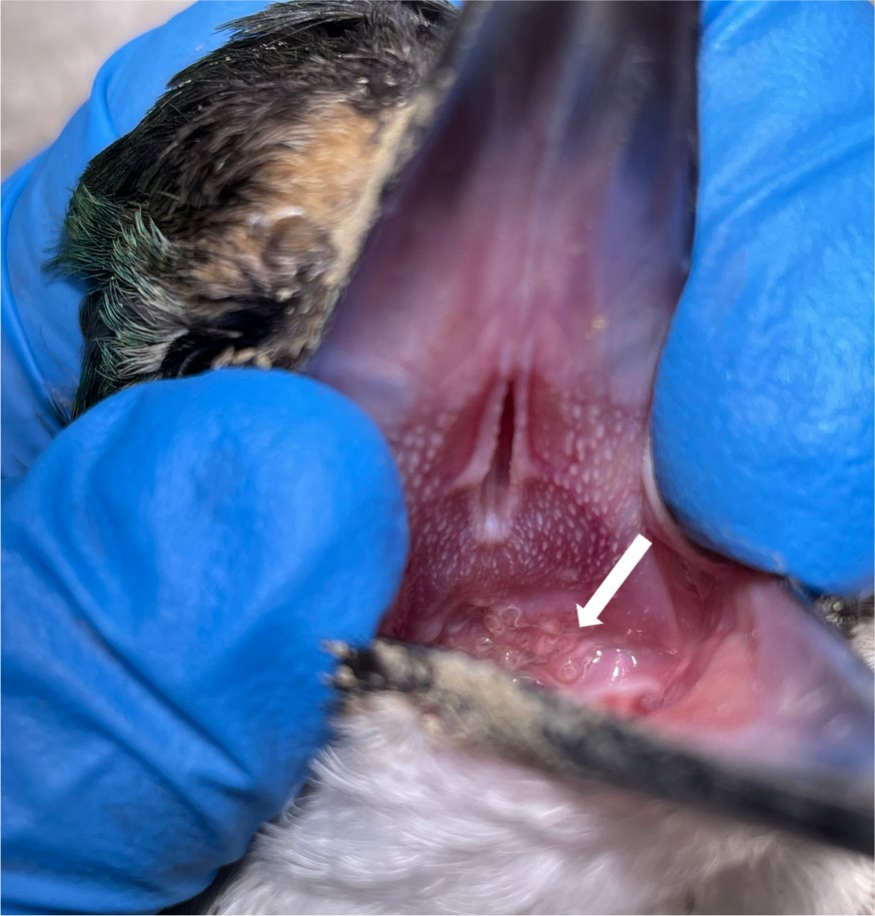
Photograph of open mouth of a sacred kingfisher showing worms embedded in the oropharyngeal mucosa (arrow).

On the basis of these morphometric differences, and particularly because of the markedly different infection site in the oral mucosa (Fig 3), the absence of previous records of *S. halcyoni* from *T. sanctus*, and the geographic isolation of the New Zealand sacred kingfisher population from other, migratory populations, we consider the present material to represent a distinct species.

**Fig. 4.**
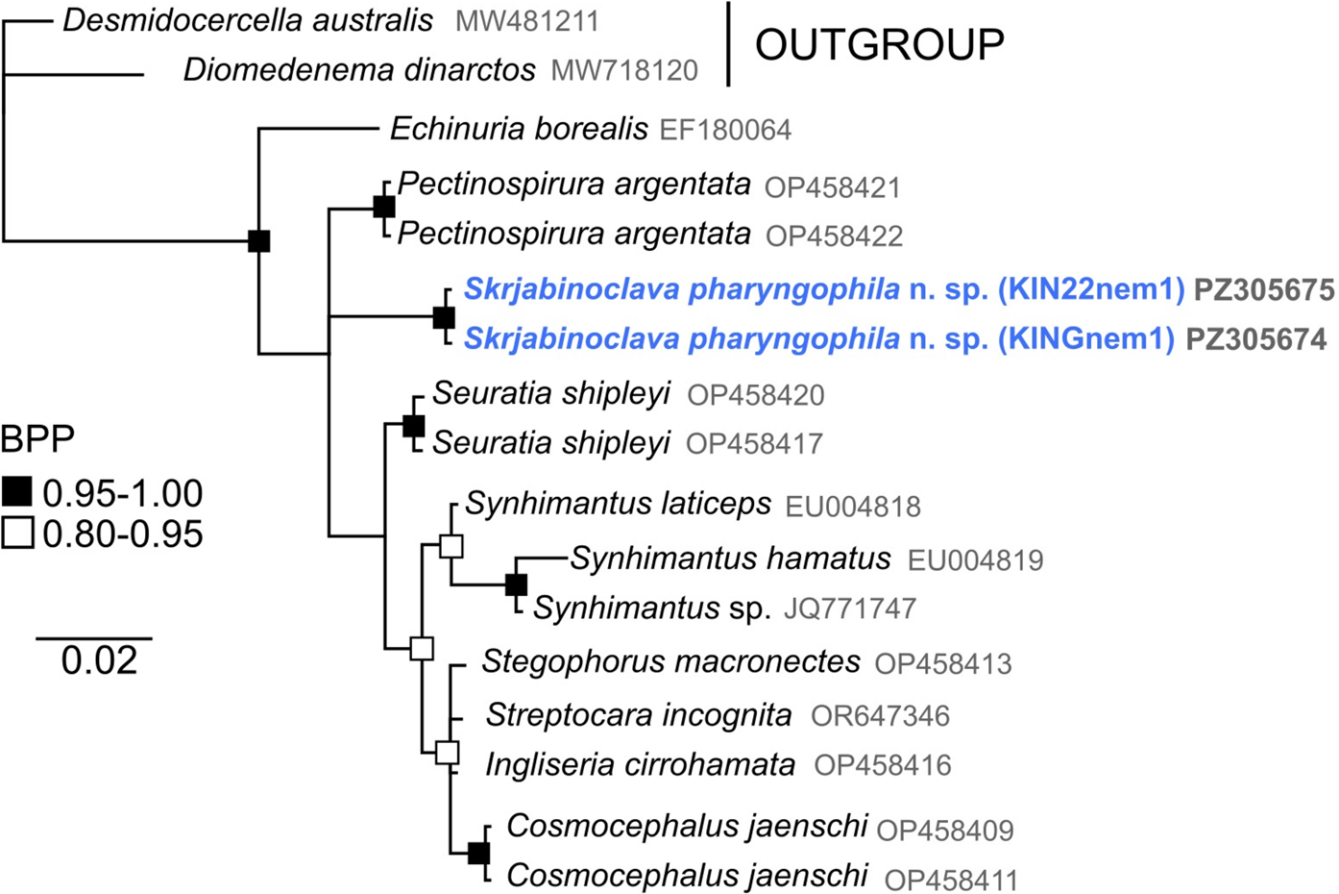
Bayesian 50% majority rule phylogenetic inference of the 18S data for species of families within superorder Spiruromorpha. Bold indicates sequences produced from this study. Bayesian Posterior Probability (BPP) illustrated by black (strongly supported, 0.95-1.0) and black-outlined (moderately supported, 0.8-0.95) squares. Scale bar indicates the number of substitutions per site.

No DNA sequences for *Skrjabinoclava* are currently available in the public domain. We sequenced the nuclear 18S (small subunit) rRNA gene (834 bp), and a BLASTn search showed the sequence to be within 2% divergence of several acuariid sequences, confirming placement within the family Acuariidae. The 18S phylogeny confirmed this positioning with high nodal support (Fig 4), although within-family resolution has limited nodal support. Mutafchiev et al. (2020), in their 28S-based phylogeny, did not sequence specimens of the genus but retrospectively placed *Skrjabinoclava* within a subclade containing *Acuaria* and *Stammerinema* based on morphological characters. They also included *Seuratia*, *Pectinospirura*, and *Echinuria* in their discussion of this subclade. However, in our 18S tree *S. pharyngophila* **n. sp.** forms a polytomy with *Pectinospirura,* and a clade including *Seuratia* and other genera. Furthermore, our phylogenetic analysis placed *Echinuria* as a sister taxa to other acuariids unlike the 28S inference of Mutafchiev et al. (2020). Based on uncorrected genetic divergence estimates of the 18S marker, *Ingliseria* and *Seuratia* had the lowest levels of divergence from *Skrjabinoclava* at 1.5% and 1.9%, respectively.

## Discussion

Of sixteen kingfishers examined from the South Island, none were infected, whereas all six birds from the North Island harboured oral nematodes. Furthermore, veterinary carers at Birdcare Aotearoa in Auckland (North Island) treated 127 kingfishers in 2025, of which 27 (21%) were infected with mouth worms, and at least eight infected birds were recorded in 2024 when records began to be kept. This marked difference in occurrence between north and south suggests that the infection may currently be restricted to the North Island, possibly reflecting the distribution of suitable intermediate hosts, which may be limited to regions with milder climatic conditions. Alternatively, the relatively sedentary behaviour of kingfishers within New Zealand may have limited dispersal of the parasite from the North Island to the South Island. Examination of kingfishers from the northernmost regions of the South Island would be of particular interest in determining whether the parasite has crossed Cook Strait.

*Skrjabinoclava pharyngophila* **n. sp.** is morphologically close to *S. halcyoni* from other kingfishers worldwide. Once available, DNA from parasites of sacred kingfishers elsewhere in their range should provide insight into the relationship between these populations and the New Zealand specimens.We suggest that the morphological differences and the shift in infection site likely arose independently in the New Zealand population after it became isolated from other populations in Australasia and Indonesia. Molecular studies indicate that allopatrically distributed, non-migratory populations of sacred kingfishers in New Caledonia and New Zealand cluster together, but remain embedded within the overall range-wide genetic diversity of the species (DeRaad et al., 2023). Given that *S. halcyoni* occurs in kingfishers elsewhere but is consistently found in the stomach or oesophagus, explanations for the morphological and ecological differences based on host physiology, immunity, or transmission dynamics seem unlikely; there is no reason to assume that these factors are greatly variable within kingfisher species. A more plausible scenario is that a chance colonisation of the oral mucosa occurred in the New Zealand population and subsequently became established. The stomach and oesphagus of all six infected birds from North Island were examined, but no nematodes were found, indicating that the worms were actively favouring the oral mucosa rather than embedding there by accident.

By definition, as they were brought to the hospital, the birds in which infections were observed had suffered trauma, although a small number of individuals were apparently uninjured but in poor condition. This makes it difficult to determine whether the presence of worms contributed to their clinical status; although adverse pathological symptoms due to the presence of worms were not observed. Removal of specimens from living birds is most effectively achieved by plucking the worms from the oral mucosa with forceps; standard anthelmintic treatments have proven ineffective so far. In some cases, the worms appear to be initially enclosed within a cyst-like structure or lesion in the buccal mucosa, from which they subsequently emerge while remaining attached by the anterior end. An interesting observation in one or two specimens was the retention of the so-called “pileus” (Fig. 2c), previously described by Bartlett (1991) in another species of *Skrjabinoclava* and in *Skrjabinocerca* Shikhobalova, 1930. The pileus was described as a transparent cap surrounding the anterior end of the worm, extending to the level of the cordons, and is thought to facilitate firm attachment to the host mucosa. The strength of this attachment is evident during removal of worms from host tissue, as considerable traction is required and specimens detach abruptly with a “snap”-like release. It is not clear whether the pileus is of parasite or host origin.

While the second intermediate host of this acuariid remains unknown, it is likely to be a crustacean. Species of *Skrjabinoclava* have been shown experimentally to utilize a range of crustacean hosts*: S. inornatae* Wong & Anderson, 1987 has been grown in fiddler crabs (*Uca* spp.), whereas *S. morrissoni* Wong & Anderson, 1987 developed in the amphipod *Corophium volutator* (Pallas) (Wong & Anderson, 1988, 1990; Wong et al., 1989). However, sacred kingfishers in New Zealand have a broad, generalist diet that includes aquatic and terrestrial invertebrates as well as lizards, small birds, and mice (McKinlay, 2013; Fitzgerald et al., 1986), so the range of potential intermediate hosts is likely to be wide and cannot presently be narrowed with confidence.

Specimens of an acuariid nematode recovered from North Island sacred kingfishers were identified as a new species of *Skrjabinoclava*, marking the first record of the genus in Australasia. This species is unique in its choice of infection site within the host and represents the first publicly available DNA sequence for the genus.

## Data Availability

Genetic sequence generated for this manuscript are deposited with Genbank for free public access.
